# Impacts of lipid-related metabolites, adiposity, and genetic background on blood eosinophil counts: the Nagahama study

**DOI:** 10.1038/s41598-021-94835-9

**Published:** 2021-07-28

**Authors:** Kenta Nishi, Hisako Matsumoto, Noriyuki Tashima, Satoru Terada, Natsuko Nomura, Mariko Kogo, Chie Morimoto, Hironobu Sunadome, Tadao Nagasaki, Tsuyoshi Oguma, Yoshinari Nakatsuka, Kimihiko Murase, Takahisa Kawaguchi, Yasuharu Tabara, Kazuhiro Sonomura, Fumihiko Matsuda, Kazuo Chin, Toyohiro Hirai

**Affiliations:** 1 Department of Respiratory Medicine, Kyoto University Graduate School of Medicine, 54 Kawaharacho, Shogoin, Sakyo-ku, Kyoto, 606-8507 Japan; 2 Department of Respiratory Care and Sleep Control Medicine, Kyoto University Graduate School of Medicine, Kyoto, Japan; 3 Center for Genomic Medicine, Kyoto University Graduate School of Medicine, Kyoto, Japan

**Keywords:** Medical research, Risk factors

## Abstract

Blood eosinophil count is a useful measure in asthma or COPD management. Recent epidemiological studies revealed that body mass index (BMI) is positively associated with eosinophil counts. However, few studies focused on the role of adiposity and fatty acid-related metabolites on eosinophil counts, including the effect of genetic polymorphism. In this community-based study involving 8265 participants (30–74 year old) from Nagahama city, we investigated the relationship between eosinophil counts and serum levels of fatty acid-related metabolites. The role of *MDC1*, a gene that is related to eosinophil counts in our previous study and encodes a protein that is thought to be involved in the repair of deoxyribonucleic acid damage, was also examined taking into account its interaction with adiposity. Serum levels of linoleic acid (LA) and β-hydroxybutyric acid (BHB) were negatively associated with eosinophil counts after adjustment with various confounders; however, there were positive interactions between serum LA and BMI and between serum BHB and BMI/body fat percentages in terms of eosinophil counts. In never-smokers, there was positive interaction for eosinophil counts between the CC genotype of *MDC1* rs4713354 and BMI/body fat percentages. In conclusion, both serum LA and BHB have negative impacts on eosinophil counts, while adiposity shows robust positive effects on eosinophil counts, partly via genetic background in never-smokers.

## Introduction

Peripheral blood eosinophil counts are one of the important clinical indices in the management of asthma and COPD^1–4^, and several epidemiological studies have been conducted to clarify factors affecting blood eosinophil counts. Eosinophil has also attracted special interest for its role in preventing obesity by Beijing of white adipose tissue in mouse models^5,6^. We and other research groups showed that higher body mass index (BMI), in addition to other factors such as allergic inflammation, male population, smoking history, and genetic backgrounds, is associated with elevated blood eosinophil counts^7–11^. As there were various reports in different cohorts that showed the association of blood eosinophil counts with factors contributing to metabolic syndrome, including lipoproteins or triglyceride (TG)^10,12–14^, the potential role of adiposity in blood eosinophil counts had been suggested. However, few studies have focused on the role of adiposity and metabolites in lipid metabolism on blood eosinophil counts^12,15^. In addition, how genetic background contributes to the association between adiposity and blood eosinophil counts remains unknown.

Free fatty acid (FFA), an important source of energy, is an intermediate product of lipid mobilization and is usually released into blood by the breakdown from TG in adipose tissue when energy demand is increased^16^. Therefore, although FFA is often elevated in obesity, the behavior of FFAs with blood eosinophil counts might be different from that of other lipoproteins and TG, yet the interrelation between FFAs and blood eosinophil counts remain unknown. In addition, butyrate, one of the short-chain fatty acids (SCFAs), and an important metabolite in obesity prevention^17,18^, is thought to be protective against type 2 inflammation and induces apoptosis of eosinophils via histone deacetylase (HDAC) inhibitory activity^19,20^. However, only small amounts of endogenous butyrate appear in peripheral blood which makes it difficult to be quantified^21^. In contrast, a ketone body β-hydroxybutyric acid (BHB), which is a derivative of butyrate^22^ and is derived from the β-oxidation of FFAs in case of glucose deficiency or impaired glucose utilization^23^, is measureable in blood. BHB is a potential candidate that might reduce blood eosinophil counts, owing to the fact that BHB also inhibits HDACs^24,25^.

Genetic backgrounds may have some effects on the association between obesity/BMI and blood eosinophil counts. *MDC1* located in 6p21, of which variant we previously identified as a potential risk gene for elevated blood eosinophil counts via genome-wide association study (GWAS) and human leukocyte antigen (HLA) imputation^10^, is involved in deoxyribonucleic acid (DNA) damage repair, due to its encoding protein which is a mediator of DNA damage checkpoint protein 1^26^. We hypothesized that a *MDC1* variant might have some effects on the association between obesity and higher blood eosinophil counts, as obesity is one of the inducers of DNA damage^27^.

In this epidemiological study, we comprehensively investigated associations between fatty acid-related metabolites and blood eosinophil counts. Interactions between obesity and genetic backgrounds for blood eosinophil counts were also explored. Should these associations exist, it would contribute largely to understanding the effects of adiposity on blood eosinophil counts.

## Results

**Figure 1:**
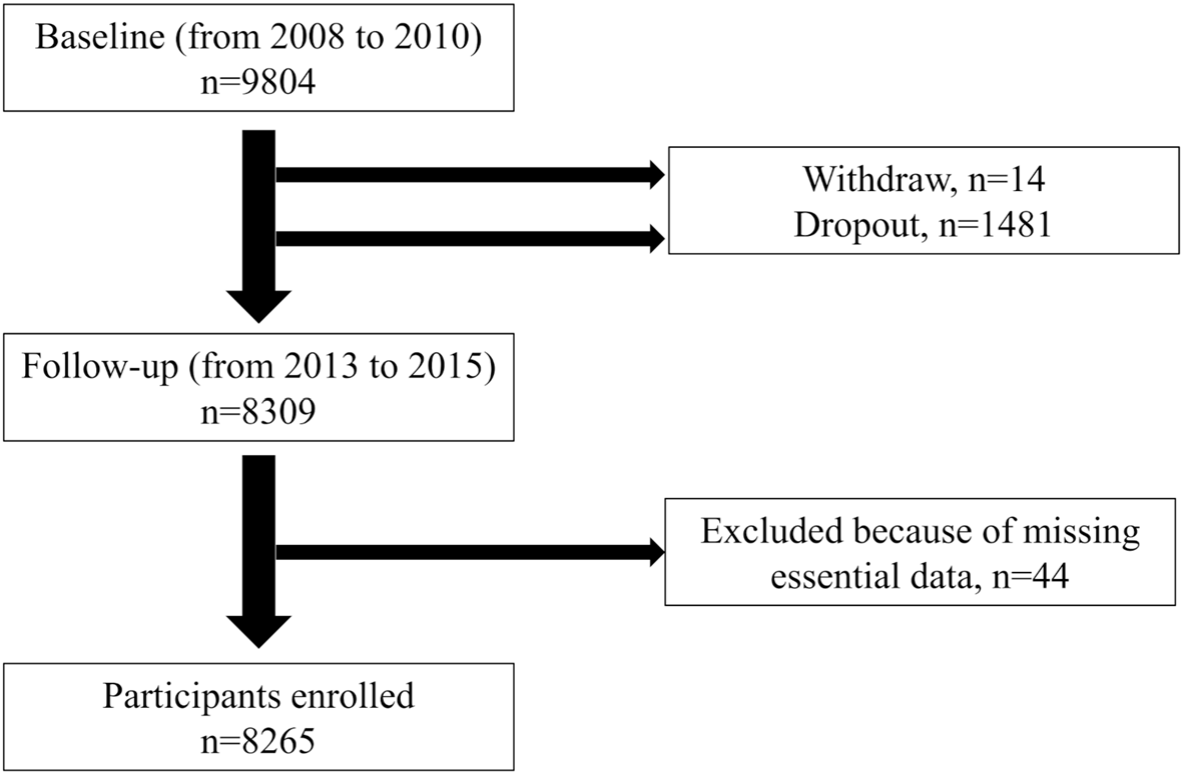
Participants flow diagram. Out of 9804 participants who were surveyed in the baseline assessment, 8265 participants were analyzed in the follow-up assessment.

A total of 8309 subjects participated in the follow-up study from November 2013 to November 2015. After exclusion of subjects with missing essential data, 8265 subjects were analyzed (Fig. 1). Table 1 shows the characteristics of the participants involved in the follow-up study.

**Table 1:** Participants’ characteristics.

	n = 8265
Age (years)	62.0 (47.0–70.0)^a^
Sex (male, n, %)	2648, 32.0%
BMI (kg/m^2^)	22.2 (3.3)^b^
Body fat percentages (%)	25.3 (7.5)^b^
Smoking (current/ex/never, %)	10.2/21.5/68.2
%FEV_1_ (%)	102.2 (16.2)^b^
Blood eosinophils (/µL)	110.0 (68.8–182.9)^a^
Blood neutrophils (/µL)	2786.4 (2242.7–3494.7)^a^
Serum total IgE (IU/mL)	65.3 (27.2–168)^a^
High-sensitivity CRP (ng/mL)	359 (175.0–762.3)^a^
Total cholesterol (mg/dL)	203.5 (33.7)^b^
LDL cholesterol (mg/dL)	117.5 (29.0)^b^
HDL cholesterol (mg/dL)	67.3 (17.4)^b^
Triglycerides (mg/dL)	79.0 (57.0–111.0)^a^
Free fatty acids (mEq/L)	0.58 (0.19)^b^

*BMI* body mass index, *FEV1* forced expiratory volume in 1 s.

^a^Median (interquartile range, IQR).

^b^Mean (SD).

### Associations between serum lipid-related metabolites and blood eosinophil counts

**Figure 2:**
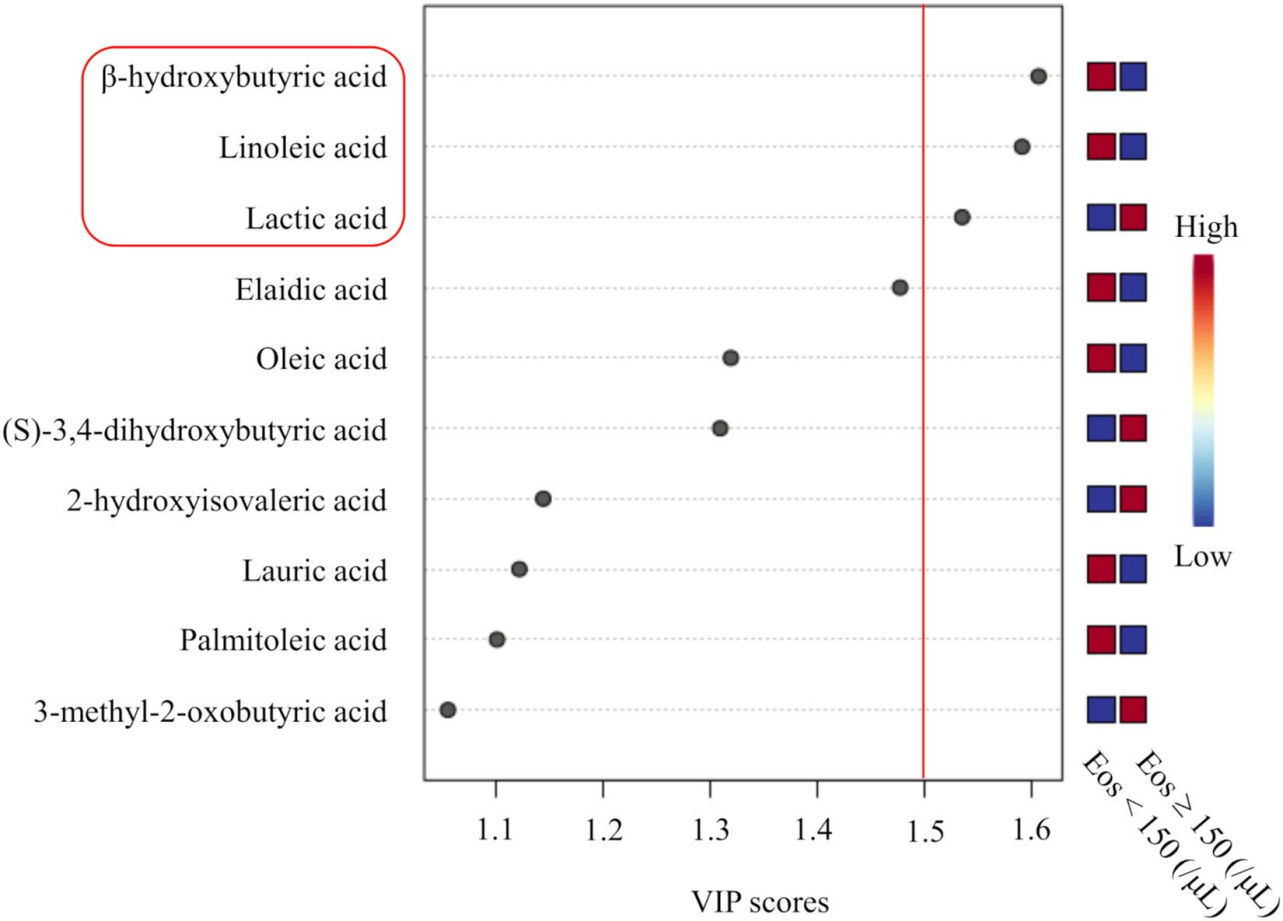
Partial least-squares discriminant analysis (PLS-DA). β-Hydroxybutyric acid (BHB) and linoleic acid (LA) were screened as candidates associated with lower blood eosinophil counts, with variable importance in projection (VIP) scores > 1.5. *Eos* blood eosinophil counts.

In the univariate analysis, higher serum levels of TG and lower HDL-C associated with higher BMI, body fat percentage, and both blood neutrophil and eosinophil counts (Supplementary Table S1). In contrast, serum FFA levels were found to show negative association with blood eosinophil counts, while showing positive association with BMI and blood neutrophil counts (Supplementary Table S1). We then analyzed contributions of metabolites in fatty acid metabolism to lower blood eosinophil counts. First, the partial least-squares discriminant analysis (PLS-DA) was performed to identify fatty acid-related metabolites associated with lower blood eosinophil counts (Fig. 2), which yielded BHB and linoleic acid (LA) with the variable importance in the projection (VIP) score over 1.5. The Pearson’s correlation coefficient between serum BHB and LA was 0.65 (*P* < 0.0001). Lactic acid, which showed a VIP score of over 1.5 for higher blood eosinophil counts, accounted for non-correlation with blood eosinophil counts as observed in the multivariate analysis (standardized partial regression coefficient, − 0.010; 95% CI − 0.033 to 0.013; *P* = 0.40).

### Clinical characteristics of subjects with higher BHB and LA

**Figure 3:**
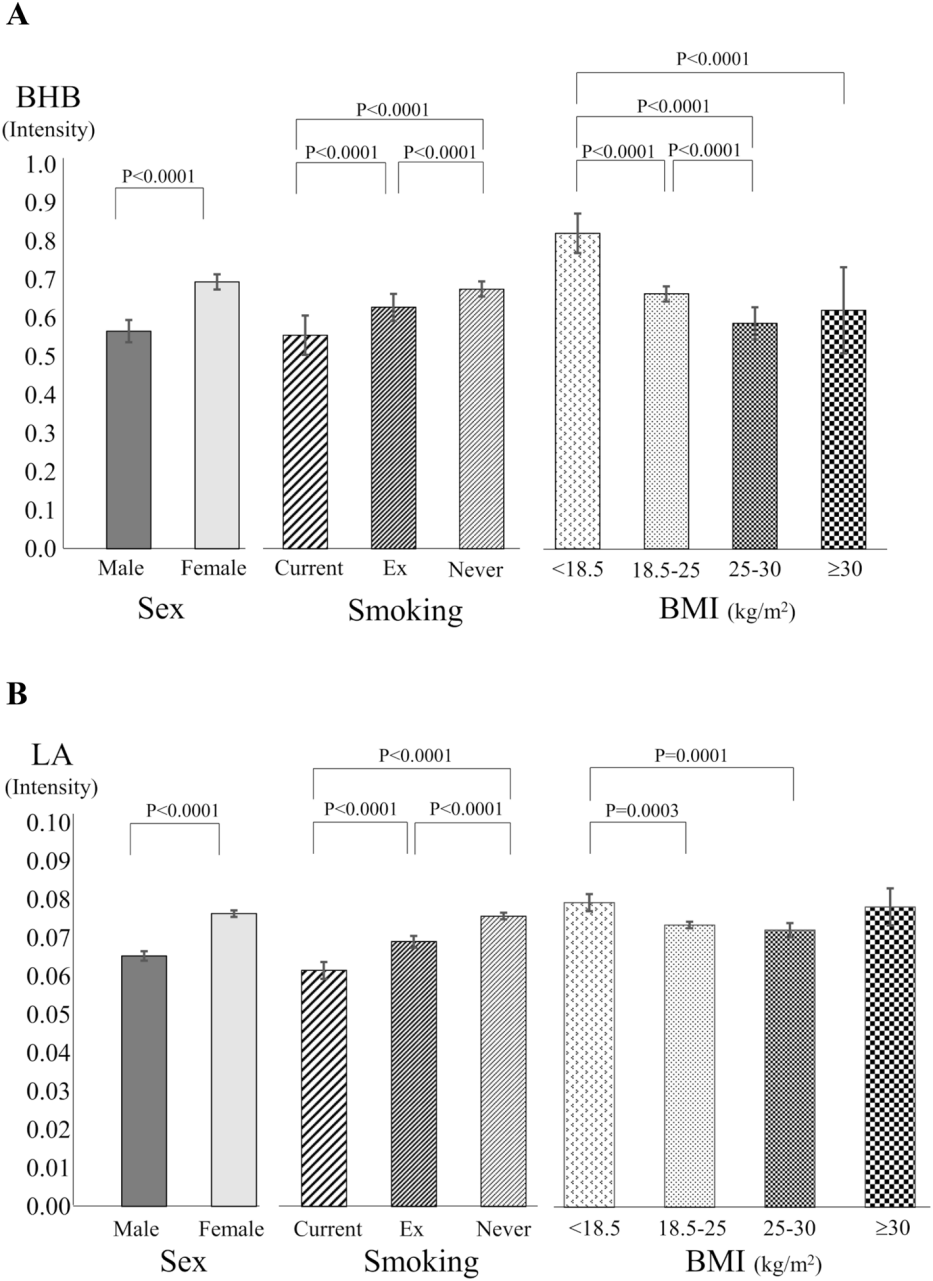
Serum levels of (**a**) BHB and (**b**) LA according to sex, smoking status, or body mass index (BMI) levels: underweight, 18.5 > BMI (kg/m^2^); normal, 25 > BMI ≥ 18.5 (kg/m^2^); overweight, 30 > BMI ≥ 25 (kg/m^2^); obese, BMI ≥ 30 (kg/m^2^). Log-transformed serum BHB and LA were used for the analysis. Means (95% confidence interval) are presented.

Clinical characteristics of subjects with higher serum BHB (Fig. 3a) and LA (Fig. 3b) were females and never-smokers. Serum BHB and LA manifested negative associations with BMI, but both serum BHB and LA levels tended to distribute in U shape against BMI—the lowest in the overweight group (BMI, 25–30 kg/m^2^) and numerically elevated in the obese population (BMI ≥ 30 kg/m^2^) (Fig. 3a,b). Serum BHB, but not LA, showed significant positive association with %FEV_1_ (standardized partial regression coefficient, 0.021; 95% CI 0.000–0.041; *P* = 0.048), after adjustment with age, sex, BMI, smoking history, blood eosinophil counts, elapsed time after a meal, and medication for dyslipidemia.

### Multivariate analysis for associations of BHB, LA, and adiposity with blood eosinophil counts

**Figure 4:**
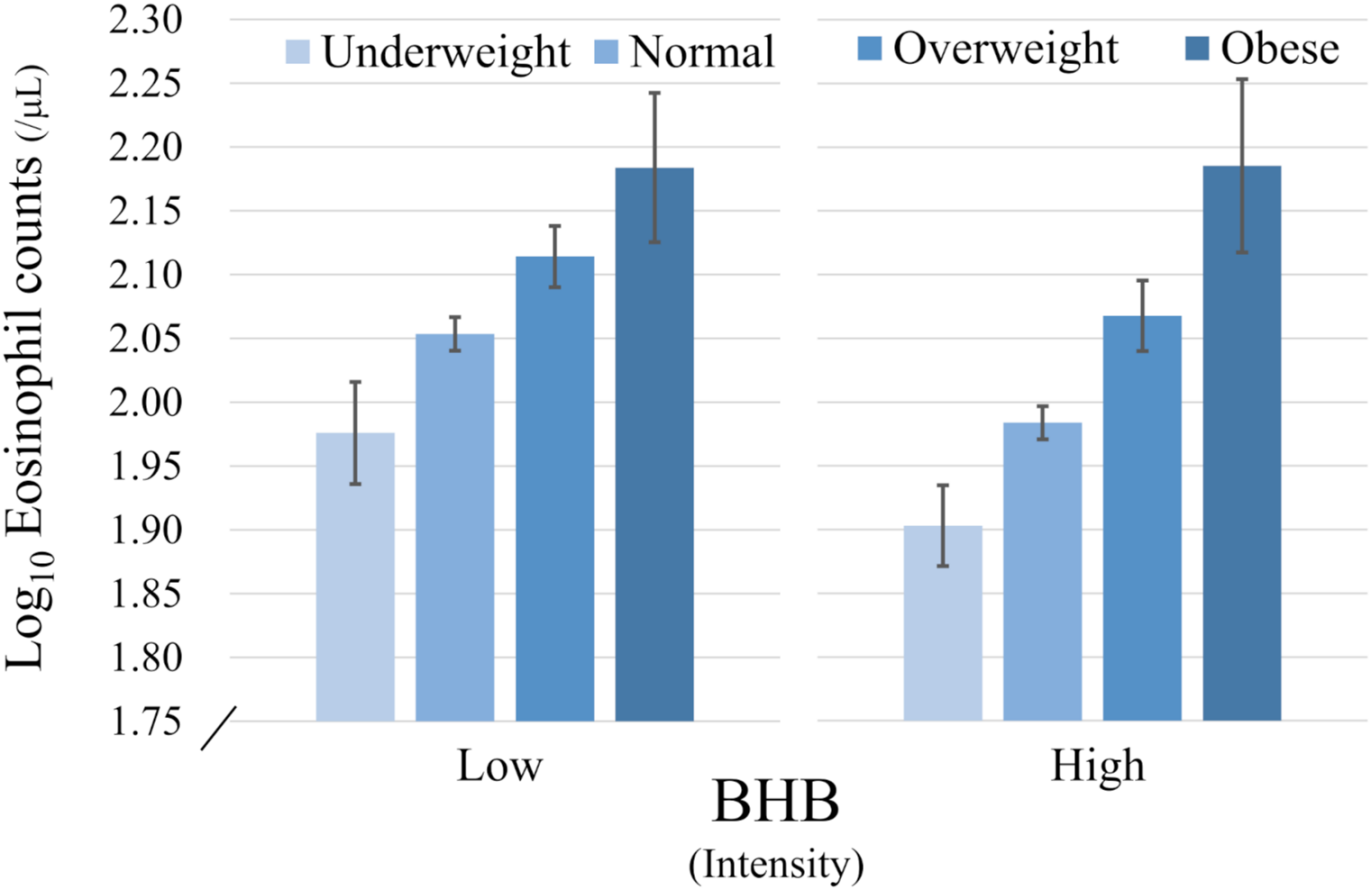
Association of blood eosinophil counts with serum BHB levels and BMI levels: underweight, 18.5 > BMI (kg/m^2^); normal, 25 > BMI ≥ 18.5 (kg/m^2^); overweight, 30 > BMI ≥ 25 (kg/m^2^); obese, BMI ≥ 30 (kg/m^2^). Subjects with log-transformed BHB < median were categorized as “low BHB” and those with log-transformed BHB ≥ median were categorized as “high BHB.” Log-transformed blood eosinophil counts and serum BHB levels were used for the analysis. Means (95% confidence interval) are presented.

In the multivariate analysis, higher serum BHB and LA were both significantly associated with lower blood eosinophil counts and higher blood neutrophil counts independent of age, sex, smoking history, elapsed time after a meal, medication for dyslipidemia, serum total IgE, HDL-C, TG, and high-sensitivity CRP (Supplementary Table S2). However, significant positive interactions for blood eosinophil counts between serum BHB and BMI (Model 1 in Table 2, Fig. 4) or body fat percentages (Model 2 in Table 2) and between serum LA and BMI (Model 1 in Table 2) were observed. There were no interactions for eosinophil counts between serum BHB or LA and sex or smoking history.

**Table 2:** Multivariate analysis for associations between serum fatty acid-related metabolites and blood eosinophil counts or neutrophil counts.

	Blood eosinophil counts^a^			Blood neutrophil counts^a^		
	β coefficient	95% CI	p value	β coefficient	95% CI	p value
**Model 1**						
BHB^a^	− 0.074	− 0.096 to − 0.052	< 0.0001	0.162	0.141–0.183	< 0.0001
BMI (kg/m^2^)	0.067	0.042–0.091	< 0.0001	0.025	0.002–0.048	0.034
BHB^a^*BMI	0.028	0.007–0.049	0.009	− 0.017	− 0.037 to 0.003	0.099
LA^a^	− 0.068	− 0.090 to − 0.045	< 0.0001	0.159	0.138–0.180	< 0.0001
BMI (kg/m^2^)	0.069	0.045–0.093	< 0.0001	0.018	− 0.005 to 0.041	0.12
LA^a^*BMI	0.028	0.007–0.049	0.009	− 0.032	− 0.052 to − 0.012	0.002
**Model 2**						
BHB^a^	− 0.077	− 0.099 to − 0.055	< 0.0001	0.161	0.140–0.182	< 0.0001
Body fat percentages (%)	0.079	0.053–0.106	< 0.0001	0.056	0.031–0.082	< 0.0001
BHB^a^*Body fat	0.028	0.007–0.049	0.010	− 0.008	− 0.028 to 0.012	0.45
LA^a^	− 0.072	− 0.094 to − 0.049	< 0.0001	0.154	0.132–0.175	< 0.0001
Body fat percentages (%)	0.085	0.058–0.112	< 0.0001	0.046	0.021–0.072	0.0004
LA^a^*Body fat	0.020	− 0.001 to 0.041	0.066	− 0.024	− 0.044 to − 0.004	0.019

*BHB* β-hydroxybutyric acid, *LA* linoleic acid, *BMI* body mass index, *β coefficient* standardized partial regression coefficient, *CI* confidence interval.

^a^Log-transformed. Multivariate analysis for blood eosinophil counts were adjusted by age, sex, smoking history, elapsed time after a meal, medication for dyslipidemia, serum HDL cholesterol, serum triglycerides, serum total IgE and high-sensitivity CRP. Multivariate analysis for blood neutrophil counts were adjusted by age, sex, smoking history, elapsed time after a meal, medication for dyslipidemia, serum HDL cholesterol, serum triglycerides and high-sensitivity CRP.

### Impact of genetic background and adiposity on blood eosinophil counts

Finally, we examined genetic effects of CC genotype of rs4713354 (*MDC1* on chromosome 6p21) which was previously identified as a potential risk factor for higher blood eosinophil counts by GWAS and HLA imputation^10^, focusing on its interaction with BMI or body fat percentages for elevated blood eosinophil counts. In the 4177 participants whom we were able to perform GWAS and HLA imputation on, there was a trend toward positive interaction between CC rs4713354 and body fat percentages for elevated blood eosinophil counts. When the analysis was confined to never-smokers (*n* = 2840; AA, AC, CC: 1771, 927, 142, resp.) to exclude the influences of smoking on elevated eosinophil counts, positive interaction for blood eosinophil counts between CC genotype and BMI (Model 1 in Table 3) or body fat percentages (Model 2 in Table 3) was significant. Analysis using LA instead of BHB yielded similar results (Table 4).

**Table 3:** Multivariate analysis in never-smokers for elevated blood eosinophil counts including BHB, BMI or body fat, rs4713354, and its interactions as variables.

	β coefficient	95% CI	p value
**Model 1**			
BHB^a^	− 0.068	− 0.107 to − 0.030	0.0005
BMI (kg/m^2^)	0.107	0.057–0.157	< 0.0001
rs4713354 AC vs AA	0.069	0.030–0.107	0.0005
CC vs AC	0.036	− 0.003 to 0.074	0.070
BMI*(AC vs AA)	− 0.026	− 0.074 to 0.021	0.27
BMI*(CC vs AC)	0.043	0.004–0.083	0.032
**Model 2**			
BHB^a^	− 0.071	− 0.109 to − 0.032	0.0003
Body fat percentages (%)	0.126	0.073–0.178	< 0.0001
rs4713354 AC vs AA	0.067	0.029–0.106	0.0006
CC vs AC	0.043	0.004–0.082	0.032
Body fat*(AC vs AA)	− 0.040	− 0.089 to 0.008	0.11
Body fat*(CC vs AC)	0.048	0.008–0.088	0.020

*BHB* β-hydroxybutyric acid, *BMI* body mass index, *β coefficient* standardized partial regression coefficient, *CI* confidence interval.

^a^Log-transformed. Adjusted by age, sex, elapsed time after a meal, medication for dyslipidemia, serum HDL cholesterol, serum triglycerides, serum total IgE and high-sensitivity CRP.

**Table 4:** Multivariate analysis in never-smokers for elevated blood eosinophil counts including LA, BMI or body fat, rs4713354, and its interactions as variables.

	β coefficient	95% CI	p value
**Model 1**			
LA^a^	− 0.062	− 0.101 to − 0.023	0.0005
BMI (kg/m^2^)	0.108	0.058–0.158	< 0.0001
rs4713354 AC vs AA	0.069	0.031–0.108	0.0005
CC vs AC	0.035	− 0.003 to 0.074	0.070
BMI*(AC vs AA)	− 0.026	− 0.073 to 0.022	0.27
BMI*(CC vs AC)	0.042	0.003–0.081	0.032
**Model 2**			
LA^a^	− 0.066	− 0.105 to − 0.027	0.0003
Body fat percentages (%)	0.131	0.079–0.184	< 0.0001
rs4713354 AC vs AA	0.068	0.029–0.107	0.0006
CC vs AC	0.043	0.004–0.082	0.032
Body fat*(AC vs AA)	− 0.041	− 0.090 to 0.007	0.11
Body fat*(CC vs AC)	0.048	0.008–0.088	0.020

*LA* linoleic acid, *BMI* body mass index, *β coefficient* standardized partial regression coefficient, *CI* confidence interval.

^a^Log-transformed. Adjusted by age, sex, elapsed time after a meal, medication for dyslipidemia, serum HDL cholesterol, serum triglycerides, serum total IgE and high-sensitivity CRP.

## Discussion

This epidemiological study is the first to demonstrate the negative associations of FFAs and its related metabolites, particularly serum levels of LA (n-6 polyunsaturated fatty acid) and BHB (ketone body), with blood eosinophil counts independent of various confounders. In never-smokers, apart from the negative impacts of FFAs on eosinophil counts, there was a positive interaction between body fat percentage and the CC genotype of *MDC1* rs4713354 in terms of blood eosinophil counts. This suggested that the subjects with the CC genotype of rs4713354 had a greater increase in blood eosinophil counts related to adiposity than those with AC/AA genotypes (Supplementary Fig. S1).

As shown in previous studies^7,12^, higher body fat percentages, higher serum levels of TG, and lower HDL were associated with elevated blood eosinophil counts and neutrophil counts, which is consistent with the findings in this study. These associations are probably due to chronic systemic inflammation inherent in adiposity^28^, such as leptin and proinflammatory cytokines^29^. In contrast, despite positive associations between serum FFA and BMI or blood neutrophil counts, serum FFA showed negative correlation with blood eosinophil counts. FFA is an intermediate product of lipid mobilization, and its serum level is determined by the balance between release from adipose tissue according to energy demand and intake into the liver and adipose tissue when not utilized as an energy source^16^. Therefore, although serum FFA levels are elevated in the obese population, similarly with TG, it is plausible that the association between blood eosinophil counts and FFA is different from that of other lipid metabolites. Indeed, serum LA, a representative long-chain fatty acid that induces systemic inflammation^31^, showed negative association with blood eosinophil counts, despite the fact that LA augments type 2 inflammation in allergic diseases as a source of arachidonic acid when present in excessive quantity^30,31^. The mechanisms underlying the negative association between serum LA and eosinophil counts remain unknown, because an in vitro study shows that LA does not affect eosinophil survival^32^. However, it is possible to speculate that LA may reduce eosinophil counts, when considering that nitrated LA activates peroxisome proliferator-activated receptor gamma (PPARγ) at nanomolar concentrations^33^, which alleviates type 2 inflammation, although LA in itself is inactive as a PPARγ ligand^34^. In addition, the negative impact of LA on eosinophil counts may reflect that of BHB, when considering that there was a moderate positive correlation between serum LA and BHB that is derived from the β-oxidation of fatty acids during the period of glucose deficiency.

Furthermore, BHB, which is increased under ketogenic conditions such as starvation or prolonged exercise^24^ and therefore is decreased in the obese population, is structurally related to SCFA^22^. As was expected, serum BHB level was negatively associated with blood eosinophil counts. A recent study reported that butyrate, which is mainly produced by the gut microbiota derived from fermentation of dietary fibers^22^, was able to attenuate activity of human eosinophils such as adhesion to the endothelium, migration, and survival. These effects were exerted probably through HDAC inhibition, and not through G protein-coupled receptor (GPR) 41 for SCFAs^20^. Another study reported that SCFA especially butyrate was able to inhibit the proliferation of group 2 innate lymphoid cells (ILC2), a critical player in inducing type 2 inflammation^35^, GATA-binding protein 3 (GATA3) expression, and interleukin-13, interleukin-5 production from ILC2 in mouse models^19^. These effects against allergic inflammation were also considered to occur via HDAC inhibitory activity of butyrate. Since BHB is also known as an HDAC inhibitor, BHB could potentially decrease survival of eosinophils^22,24^.

This epidemiological study confirmed that various factors yield positive impacts on blood eosinophil counts as previously demonstrated^7,36^. For example, although we found negative impacts of serum BHB and LA on blood eosinophil counts, these were attenuated by higher BMI and body fat percentages, as shown by positive interactions for blood eosinophil counts. Additionally, as observed in this study, males and smokers, which are known to be factors for higher blood eosinophil counts in the general population^7,10^, showed lower serum levels of BHB and LA than their counterparts, which is a similar finding of previous studies^37–39^. Thus males, smoking, and lower serum BHB or LA may additively increase blood eosinophil counts.

Genetic backgrounds may also intervene in the robust association between adiposity and elevated blood eosinophil counts. *GATA2*, *IL1RL1*, and *IL5* are known risk genes for elevated blood eosinophil counts, and proteins encoded by them are well-known factors in type 2 inflammation^11^. Differently from these genes, *MDC1* encodes a mediator of DNA damage checkpoint protein 1, which plays an important role in DNA damage response by recruiting a number of repair proteins to the damaged sites^26^. *MDC1* rs4713354 which is located in the promoter region of *MDC1* is a known functional polymorphism. A to C transversion of rs4713354 causes a loss of transcription factor binding sites, which may lead to the decrease of expression of *MDC1*^40^. In the analysis of radiation-induced apoptosis in A549 cells, which are human lung adenocarcinoma-derived epithelial-like cells, apoptosis was largely suppressed with *MDC1* small interfering RNA, which was restored with addition of *MDC1*^41^. Thus, decreased expression of *MDC1* via A to C transversion of rs4713354 may enhance survival of eosinophils that have DNA repair machinery^42^ in the milieu of oxidative DNA damage in obesity^27^. Therefore, we speculated that effects of *MDC1* rs4713354 variants may be evident in the obese population. Indeed, there were positive interactions for blood eosinophil counts between CC genotype of rs4713354 and BMI or body fat percentages after excluding the influences of smoking on blood eosinophil counts. These findings suggest that subjects with CC genotype of rs4713354 were susceptible to the effects of adiposity on elevated blood eosinophil counts.

Lastly, apart from eosinophil counts, the association of serum BHB with %FEV_1_ is elaborated. Serum BHB, but not LA, correlated with higher %FEV_1_ after adjustment with BMI and other factors, suggesting that BHB might relax airway smooth muscle. SCFAs like butyrate may cause contraction of airway smooth muscle by activating GPR41^43^. Meanwhile, a study reported that water-soluble BHB acts as antagonist of GPR41 at the level of sympathetic ganglion^44^. When considering the capability of butyrate for contractions of airway smooth muscle, it is possible to speculate that relaxing of airway smooth muscle might be induced by BHB through GPR41 inhibition, although this requires further evidence.

Although butyrate could have strong association with eosinophil apoptosis^20^, neither fecal nor serum butyrate was available in this study, which is one of the considered limitations of this study, since no stool sample was obtained and serum butyrate was difficult to measure. However, serum BHB was examined. Studies focusing on beneficial effects of butyrate and BHB on eosinophilic inflammation may be prompted by the negative impacts of BHB on blood eosinophil counts observed in this study. Another limitation was the limited investigation of the interactions between BMI and other risk genes for elevated blood eosinophils^11^: *GATA2*, *IL1RL1*, and *IL5*. In addition, this interaction of *MDC1* was not validated in the second population. Nonetheless, the positive interaction for blood eosinophil counts between the variant of *MDC1* and BMI/body fat percentages in never-smokers may shed light on the involvement of DNA damage and repair of eosinophils from the perspective of adiposity.

In conclusion, serum FFA and its metabolite, particularly LA and BHB, may decrease blood eosinophil counts, while adiposity increases blood eosinophil counts synergistically with genetic background in never-smokers. These may suggest that the balance of adiposity and its breakdown may affect blood eosinophil counts.

## Methods

### Study design and participants

The Nagahama Prospective Genome Cohort for Comprehensive Human Bioscience (The Nagahama Study) is a community-based prospective cohort study^45,46^. The participants were recruited from generally healthy residents, aged 30–74 years, in Nagahama City, Shiga Prefecture, Japan. A total of 9804 subjects were enrolled from 2008 to 2010, considering the inclusive period the baseline assessment of the Nagahama Study. After 5 years from the baseline assessment, a follow-up survey was conducted from November 2013 to November 2015 on subjects who had not waived their consent. In the present study, subjects were excluded if they had missing essential data of blood eosinophil counts or metabolites. This study obtained approval from the Ethics Committee of Kyoto University Graduate School of Medicine and the Nagahama Municipal Review Board. All methods were performed in accordance with the principles of the Declaration of Helsinki. Written informed consent was obtained from all participants.

### Questionnaire and measurements

Clinical measurements were composed of self-reported questionnaires, pulmonary function tests, and blood sampling. Asthma and medication for dyslipidemia was determined by self-reported questionnaire. Next, pulmonary function was measured by a computed spirometer with automated quality checks (SP-350 COPD; Fukuda Denshi, Tokyo, Japan). Aside from serum total IgE, which was measured at baseline assessment only, blood data at follow-up assessment were analyzed. The time elapsed after their last meal was also recorded. Gas chromatography–mass spectrometry (GC–MS) analysis was conducted using GCMS-QP2010 Ultra (Shimadzu, Kyoto, Japan) at follow-up assessment according to the previous study^47^. In brief, water, methanol, chloroform, and the internal standard solution (2-isopropylmalic acid) were added to each serum sample. The supernatant after centrifugation was dried and was processed under trimethylsilyl derivatization. Then, the extract was subjected to GC–MS analysis. For data acquisition and processing, GC–MS solution software version 2.71 (Shimadzu, Kyoto, Japan) was used. Metabolites were identified using the Shimadzu GC/MS database or by comparison with authentic standards. The peak area of each metabolite was calculated and normalized.

For genotyping and GWAS, DNA was extracted from blood samples for analysis. The HLA imputation consisted of 4615 variants in total: 3848 single nucleotide polymorphisms (SNPs), 645 HLA amino acids, 53 two-digit HLA alleles, and 69 four-digit HLA alleles. A detailed description of GWAS and HLA imputation is shown in our previous article^10^.

### Statistical analysis

PLS-DA was performed using MetaboAnalyst 4.0^48^. Subsequent to log-transformation, 24 lipid-related metabolite data (Supplementary Table S3) were mean-centered and divided by the standard deviation of each variable. Then, metabolites with lower blood eosinophil counts were observed using the PLS-DA. The other statistical analyses were performed with JMP version Pro15 (SAS institute Inc., Tokyo, Japan). Pearson’s correlation coefficient was used to examine correlation between two variables. One-way analysis of variance with post hoc Tukey’s Honest Significant Difference test or Student’s *t* test was performed to conduct comparisons in between groups. Next, multivariate regression analyses were performed to identify factors associated with blood eosinophil counts at follow-up assessment. *P* values of less than 0.05 were regarded as statistically significant.

### Supplementary Information

Supplementary Information.

Supplementary Figure S1.

## Data Availability

The data analyzed during the current study are not publicly available because we did not obtain prior consent from the participants, but are available from the corresponding author on reasonable request.

## Appendix

The Nagahama study group executive committee comprises the following individuals: Yasuharu Tabara, Takahisa Kawaguchi, Kazuya Setoh, Yoshimitsu Takahashi, Shinji Kosugi, Takeo Nakayama, and Fumihiko Matsuda from the Center for Genomic Medicine, Kyoto University Graduate School of Medicine (YaT, TK, KS, FM); Department of Health Informatics (YoT, TN); Department of Medical Ethics and Medical Genetics (SK), Kyoto University School of Public Health.
