# Supplementary material for: Impacts of lipid-related metabolites, adiposity, and genetic background on blood eosinophil counts: the Nagahama study
Source: Sci Rep. 2021 Jul 28;11:15373. doi: 10.1038/s41598-021-94835-9 (PMC8319143; doi:10.1038/s41598-021-94835-9)
Supplement: Supplementary file 1 — Supplementary Information. [file 41598_2021_94835_MOESM1_ESM.docx]

**Impacts of lipid-related metabolites, adiposity, and genetic background on blood eosinophil counts: The Nagahama study**

**Authors**

Kenta Nishi^1^, Hisako Matsumoto^1,*^, Noriyuki Tashima^1^, Satoru Terada^1^, Natsuko Nomura^1^, Mariko Kogo^1^, Chie Morimoto^1^, Hironobu Sunadome^2^, Tadao Nagasaki^2^, Tsuyoshi Oguma^1^, Yoshinari Nakatsuka^2^, Kimihiko Murase^2^, Takahisa Kawaguchi^3^, Yasuharu Tabara^3^, Kazuhiro Sonomura^3^, Fumihiko Matsuda^3^, Kazuo Chin^2^, Toyohiro Hirai^1^

^1^Department of Respiratory Medicine, Kyoto University Graduate School of Medicine, Kyoto, Japan.

^2^Department of Respiratory Care and Sleep Control Medicine, Kyoto University Graduate School of Medicine, Kyoto, Japan.

^3^Center for Genomic Medicine, Kyoto University Graduate School of Medicine, Kyoto, Japan.

**Corresponding Author**

Hisako Matsumoto, MD, PhD, Department of Respiratory Medicine, Kyoto University Graduate School of Medicine, 54 Kawaharacho, Shogoin, Sakyo-ku, Kyoto 606-8507, Japan.

E-mail: matsumoto.hisako.v92@kyoto-u.jp

**Supplementary information**

**Legend of Supplementary Fig. S1**

Schematic image showing a positive interaction between the CC genotype of rs4713354 and adiposity in terms of blood eosinophil counts. There was a significant difference between the slopes of the two lines; subjects with CC genotype showed a greater increase in blood eosinophil counts as related to adiposity than those with AC/AA genotypes. The solid line and filled circles represent CC genotype, whereas the dotted line and open circles represent AC/AA genotypes.

Supplementary Table S1. Univariate analysis for associations between serum lipid-related metabolites and BMI, body fat percentages, blood eosinophil counts, or blood neutrophil counts.

|  | BMI (kg/m^2^) | | |  | Body fat percentages (%) | | |
| --- | --- | --- | --- | --- | --- | --- | --- |
|  | β coefficient | 95% CI | P value |  | β coefficient | 95% CI | P value |
| HDL cholesterol (mg/dL) | −0.383 | −0.403 to −0.363 | <0.0001 |  | −0.179 | −0.200 to −0.157 | <0.0001 |
| Triglycerides^†^ (mg/dL) | 0.381 | 0.361 to 0.401 | <0.0001 |  | 0.240 | 0.219 to 0.261 | <0.0001 |
| Free fatty acids (mEq/L) | 0.040 | 0.019 to 0.062 | 0.0002 |  | 0.174 | 0.152 to 0.195 | <0.0001 |

BMI, body mass index; β coefficient, standardized partial regression coefficient; CI, confidence interval

|  | Blood eosinophil counts^†^ | | |  | Blood neutrophil counts^†^ | | |
| --- | --- | --- | --- | --- | --- | --- | --- |
|  | β coefficient | 95% CI | P value |  | β coefficient | 95% CI | P value |
| HDL cholesterol (mg/dL) | −0.161 | −0.182 to −0.140 | <0.0001 |  | −0.189 | −0.210 to −0.167 | <0.0001 |
| Triglycerides^†^ (mg/dL) | 0.150 | 0.128 to 0.171 | <0.0001 |  | 0.196 | 0.175 to 0.218 | <0.0001 |
| Free fatty acids (mEq/L) | −0.094 | −0.115 to −0.072 | <0.0001 |  | 0.166 | 0.145 to 0.187 | <0.0001 |

β coefficient, standardized partial regression coefficient; CI, confidence interval

^†^Log-transformed.

Supplementary Table S2. Full results of multivariate analysis for associations between BHB/LA and blood eosinophil or neutrophil counts

| Model 1 | Blood eosinophil counts^†^ | | |  | Blood neutrophil counts^†^ | | |
| --- | --- | --- | --- | --- | --- | --- | --- |
|  | β coefficient | 95% CI | P value |  | β coefficient | 95% CI | P value |
| BHB^†^ | −0.074 | −0.096 to −0.052 | <0.0001 |  | 0.162 | 0.141 to 0.183 | <0.0001 |
| BMI (kg/m^2^) | 0.067 | 0.042 to 0.091 | <0.0001 |  | 0.025 | 0.002 to 0.048 | 0.034 |
| BHB^†^*BMI | 0.028 | 0.007 to 0.049 | 0.009 |  | −0.017 | −0.037 to 0.003 | 0.099 |
| Age (years) | −0.057 | −0.080 to −0.033 | <0.0001 |  | −0.117 | −0.139 to −0.095 | <0.0001 |
| Sex (male) | 0.064 | 0.036 to 0.092 | <0.0001 |  | 0.039 | 0.012 to 0.065 | 0.004 |
| Smoking (never) | Reference |  | 1 |  | Reference |  | 1 |
| Smoking (ex) | −0.035 | −0.068 to −0.002 | 0.040 |  | −0.066 | −0.097 to −0.034 | <0.0001 |
| Smoking (current) | 0.083 | 0.050 to 0.116 | <0.0001 |  | 0.168 | 0.137 to 0.199 | <0.0001 |
| Serum total IgE (IU/mL) | 0.167 | 0.146 to 0.189 | <0.0001 |  |  |  |  |
| High-sensitivity CRP^†^ (ng/mL) | 0.050 | 0.027 to 0.073 | <0.0001 |  | 0.257 | 0.235 to 0.279 | <0.0001 |
| HDL cholesterol (mg/dL) | −0.042 | −0.068 to −0.016 | 0.002 |  | −0.053 | −0.077 to −0.028 | <0.0001 |
| Triglycerides^†^ (mg/dL) | 0.042 | 0.016 to 0.069 | 0.002 |  | 0.112 | 0.087 to 0.137 | <0.0001 |
| elapsed time after a meal (hours) | −0.033 | −0.054 to −0.011 | 0.003 |  | −0.081 | −0.101 to −0.061 | <0.0001 |
| medication for dyslipidemia (yes) | 0.040 | 0.018 to 0.063 | 0.0004 |  | 0.017 | −0.004 to 0.039 | 0.11 |
|  |  | |  |  |  | |  |
|  | β coefficient | 95% CI | P value |  | β coefficient | 95% CI | P value |
| LA^†^ | −0.068 | −0.090 to −0.045 | <0.0001 |  | 0.159 | 0.138 to 0.180 | <0.0001 |
| BMI (kg/m^2^) | 0.069 | 0.045 to 0.093 | <0.0001 |  | 0.018 | −0.005 to 0.041 | 0.12 |
| LA^†^*BMI | 0.028 | 0.007 to 0.049 | 0.009 |  | −0.032 | −0.052 to −0.012 | 0.002 |
| Age (years) | −0.050 | −0.073 to −0.026 | <0.0001 |  | −0.131 | −0.153 to −0.109 | <0.0001 |
| Sex (male) | 0.062 | 0.033 to 0.090 | <0.0001 |  | 0.046 | 0.020 to 0.073 | 0.0006 |
| Smoking (never) | Reference |  | 1 |  | Reference |  | 1 |
| Smoking (ex) | −0.035 | −0.068 to −0.002 | 0.038 |  | −0.066 | −0.097 to −0.034 | <0.0001 |
| Smoking (current) | 0.082 | 0.049 to 0.115 | <0.0001 |  | 0.171 | 0.139 to 0.202 | <0.0001 |
| Serum total IgE (IU/mL) | 0.167 | 0.146 to 0.189 | <0.0001 |  |  |  |  |
| High-sensitivity CRP^†^ (ng/mL) | 0.046 | 0.023 to 0.069 | 0.0001 |  | 0.266 | 0.244 to 0.288 | <0.0001 |
| HDL cholesterol (mg/dL) | −0.031 | −0.058 to −0.005 | 0.021 |  | −0.078 | −0.104 to −0.053 | <0.0001 |
| Triglycerides^†^ (mg/dL) | 0.063 | 0.037 to 0.090 | <0.0001 |  | 0.064 | 0.039 to 0.089 | <0.0001 |
| elapsed time after a meal (hours) | −0.030 | −0.051 to −0.008 | 0.006 |  | −0.088 | −0.109 to −0.068 | <0.0001 |
| medication for dyslipidemia (yes) | 0.040 | 0.017 to 0.062 | 0.0005 |  | 0.019 | −0.003 to 0.040 | 0.085 |

BHB, β-hydroxybutyric acid; LA, linoleic acid; BMI, body mass index; β coefficient, standardized partial regression coefficient; CI, confidence interval

| Model 2 | Blood eosinophil counts^†^ | | |  | Blood neutrophil counts^†^ | | |
| --- | --- | --- | --- | --- | --- | --- | --- |
|  | β coefficient | 95% CI | P value |  | β coefficient | 95% CI | P value |
| BHB^†^ | −0.077 | −0.099 to −0.055 | <0.0001 |  | 0.161 | 0.140 to 0.182 | <0.0001 |
| Body fat percentages (%) | 0.079 | 0.053 to 0.106 | <0.0001 |  | 0.056 | 0.031 to 0.082 | <0.0001 |
| BHB^†^*Body fat | 0.028 | 0.007 to 0.049 | 0.010 |  | −0.008 | −0.028 to 0.012 | 0.45 |
| Age (years) | −0.062 | −0.085 to −0.038 | <0.0001 |  | −0.123 | −0.145 to −0.101 | <0.0001 |
| Sex (male) | 0.108 | 0.077 to 0.140 | <0.0001 |  | 0.071 | 0.042 to 0.101 | <0.0001 |
| Smoking (never) | Reference |  | 1 |  | Reference |  | 1 |
| Smoking (ex) | −0.037 | −0.071 to −0.004 | 0.028 |  | −0.070 | −0.102 to −0.039 | <0.0001 |
| Smoking (current) | 0.085 | 0.052 to 0.118 | <0.0001 |  | 0.172 | 0.140 to 0.203 | <0.0001 |
| Serum total IgE (IU/mL) | 0.166 | 0.144 to 0.188 | <0.0001 |  |  |  |  |
| High-sensitivity CRP^†^ (ng/mL) | 0.046 | 0.022 to 0.069 | 0.0001 |  | 0.248 | 0.226 to 0.270 | <0.0001 |
| HDL cholesterol (mg/dL) | −0.044 | −0.070 to −0.018 | 0.001 |  | −0.049 | −0.073 to −0.024 | 0.0001 |
| Triglycerides^†^ (mg/dL) | 0.038 | 0.012 to 0.065 | 0.005 |  | 0.107 | 0.082 to 0.132 | <0.0001 |
| elapsed time after a meal (hours) | −0.035 | −0.056 to −0.013 | 0.002 |  | −0.084 | −0.104 to −0.064 | <0.0001 |
| medication for dyslipidemia (yes) | 0.039 | 0.017 to 0.062 | 0.0007 |  | 0.015 | −0.006 to 0.037 | 0.16 |
|  |  | |  |  |  | |  |
|  | β coefficient | 95% CI | P value |  | β coefficient | 95% CI | P value |
| LA^†^ | −0.072 | −0.094 to −0.049 | <0.0001 |  | 0.154 | 0.132 to 0.175 | <0.0001 |
| Body fat percentages (%) | 0.085 | 0.058 to 0.112 | <0.0001 |  | 0.046 | 0.021 to 0.072 | 0.0004 |
| LA^†^*Body fat | 0.020 | −0.001 to 0.041 | 0.066 |  | −0.024 | −0.044 to −0.004 | 0.019 |
| Age (years) | −0.057 | −0.081 to −0.033 | <0.0001 |  | −0.135 | −0.157 to −0.112 | <0.0001 |
| Sex (male) | 0.109 | 0.077 to 0.140 | <0.0001 |  | 0.074 | 0.044 to 0.103 | <0.0001 |
| Smoking (never) | Reference |  | 1 |  | Reference |  | 1 |
| Smoking (ex) | −0.038 | −0.071 to −0.004 | 0.027 |  | −0.071 | −0.102 to −0.039 | <0.0001 |
| Smoking (current) | 0.085 | 0.051 to 0.118 | <0.0001 |  | 0.174 | 0.142 to 0.205 | <0.0001 |
| Serum total IgE (IU/mL) | 0.166 | 0.144 to 0.187 | <0.0001 |  |  |  |  |
| High-sensitivity CRP^†^ (ng/mL) | 0.041 | 0.017 to 0.064 | 0.0007 |  | 0.258 | 0.235 to 0.280 | <0.0001 |
| HDL cholesterol (mg/dL) | −0.032 | −0.058 to −0.005 | 0.019 |  | −0.074 | −0.099 to −0.049 | <0.0001 |
| Triglycerides^†^ (mg/dL) | 0.060 | 0.033 to 0.086 | <0.0001 |  | 0.061 | 0.036 to 0.086 | <0.0001 |
| elapsed time after a meal (hours) | −0.031 | −0.053 to −0.010 | 0.004 |  | −0.091 | −0.111 to −0.071 | <0.0001 |
| medication for dyslipidemia (yes) | 0.038 | 0.016 to 0.061 | 0.0008 |  | 0.017 | −0.004 to 0.038 | 0.12 |

BHB, β-hydroxybutyric acid; LA, linoleic acid; β coefficient, standardized partial regression coefficient; CI, confidence interval ^†^Log-transformed. Multivariate analysis for blood eosinophil counts were adjusted by age, sex, smoking history, elapsed time after a meal, medication for dyslipidemia, serum HDL cholesterol, serum triglycerides^†^, serum total IgE^†^ and high-sensitivity CRP^†^. Multivariate analysis for blood neutrophil counts were adjusted by age, sex, smoking history, elapsed time after a meal, medication for dyslipidemia, serum HDL cholesterol, serum triglycerides^†^ and high-sensitivity CRP^†^.

Supplementary Table S3. Fatty acid-related metabolites on which the partial least-squares discriminant analysis was performed.

| Lactic acid |
| --- |
| Succinic acid |
| Caproic acid |
| Octanoic acid |
| Decanoic acid |
| Lauric acid |
| Myristic acid |
| Palmitoleic acid |
| Margaric acid |
| Oleic acid |
| Elaidic acid |
| Linoleic acid |
| 2-Hydroxyisovaleric acid |
| 3-Hydroxyisovaleric acid |
| 2-Hydroxybutyric acid |
| 3-Hydroxybutyric acid (β-Hydroxybutyric acid) |
| 2-Hydroxyisobutyric acid |
| 3-Hydroxyisobutyric acid |
| 3,4-Dihydroxybutyric acid |
| Acetoacetic acid |
| 2-Oxobutyric acid |
| 3-Methyl-2-oxobutyric acid |
| 3-Methyl-2-oxovaleric acid |
| 2-Oxoisocaproic acid |
