# Supplementary figures and images for: Impacts of lipid-related metabolites, adiposity, and genetic background on blood eosinophil counts: the Nagahama study
Source: Sci Rep. 2021 Jul 28;11:15373. doi: 10.1038/s41598-021-94835-9 (PMC8319143; doi:10.1038/s41598-021-94835-9)

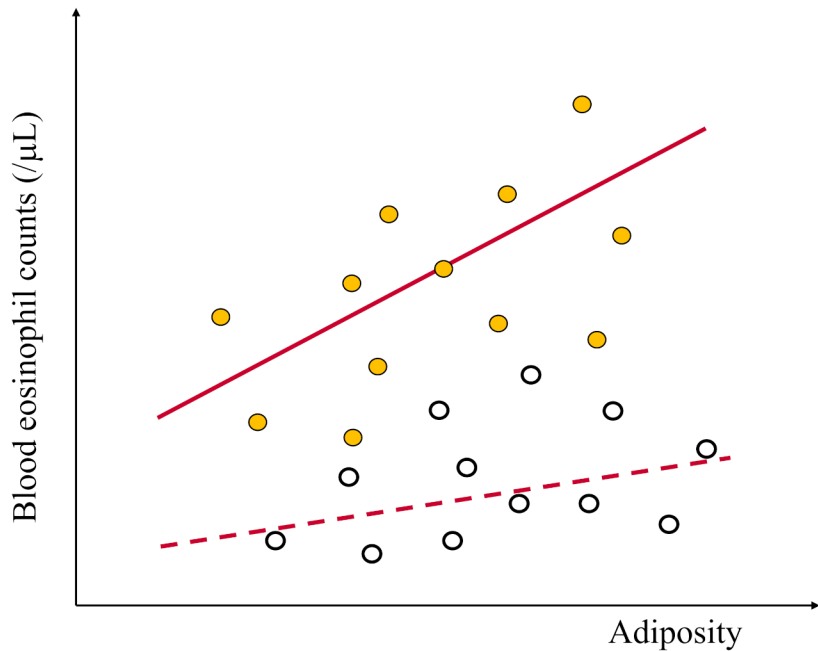

Supplementary Fig. S1

Supplement: Supplementary file 2 — Supplementary Figure S1. [file 41598_2021_94835_MOESM2_ESM.pdf]
